# Effects of *Wolbachia* elimination and B-vitamin supplementation on bed bug development and reproduction

**DOI:** 10.1038/s41598-022-14505-2

**Published:** 2022-06-17

**Authors:** Mauri L. Hickin, Madhavi L. Kakumanu, Coby Schal

**Affiliations:** Department of Entomology and Plant Pathology, North Carolina State University, Raleigh, NC USA

**Keywords:** Evolution, Microbiology, Physiology, Entomology

## Abstract

Obligate blood feeders, such as *Cimex lectularius* (common bed bug), have symbiotic associations with nutritional endosymbionts that produce B-vitamins. To quantify the symbiont’s contribution to host fitness in these obligate mutualisms, the symbiont must be eliminated and its absence rigorously confirmed. We developed and validated procedures for complete elimination of *Wolbachia* (*Wb*) in bed bugs and quantified development and reproduction in bed bugs with and without *Wb* and with and without B-vitamins supplementation. Aposymbiotic bed bugs had slower nymphal development, reduced adult survivorship, smaller adult size, fewer eggs per female, and lower hatch rate than bed bugs that harbored *Wb*. In aposymbiotic bed bugs that were fed B-vitamins-supplemented blood, nymph development time, adult survivorship and hatch rate recovered to control levels, but adult size and egg number only partially recovered. These results underscore the nutritional dependence of bed bugs on their *Wb* symbiont and suggest that *Wb* may provide additional nutritional benefits beyond the B-vitamin mix that we investigated.

## Introduction

More than 7400 species of insects, arachnids, leeches, crustaceans and vampire bats are obligate blood feeders^1,2^. Some of the insects include Anaplura (sucking lice) with more than 5000 species, Hemiptera (true bugs) with at least 262 species, and Diptera (true flies) with more than 650 species^2^. Although blood is a readily available resource, it is nutritionally unbalanced and contains noxious elements, which obligate blood feeders must overcome. Although vertebrate blood is high in protein, it is low in carbohydrates, lipids, and vitamins, and heme and iron require effective detoxification pathways^3,4^. Insects cannot synthesize eight essential B-vitamins that function as co-enzymes in metabolic processes, and most insects acquire B-vitamins from food. However, blood generally contains low concentrations of B-vitamins, and obligate blood feeders overcome this deficit through mutualistic associations with nutritional endosymbionts that produce B-vitamins. Well-investigated examples include *Wigglesworthia* endosymbionts in tsetse flies, *Glossina* spp. (Diptera)^5^ and *Nocardia rhodnii* in the kissing bug, *Rhodnius prolixus* (Hemiptera)^6,7^. Interestingly, the B-vitamin biosynthesis pathways in each symbiont and the B-vitamins they produce vary depending on the insect-endosymbiont system^2^. Moreover, the amount of each B-vitamin required for various insect species is poorly understood^8^, leaving a large gap in knowledge about vitamin requirements in insects, and especially in blood feeders.

The common bed bug, *Cimex lectularius* (Hemiptera), is an obligate blood feeder. Whereas some blood-feeders, like flies, sand flies, and mosquitoes, obtain nutrients from other sources during larval stages (e.g., flesh, detritus) or as adults (e.g., nectar), all mobile life stages of the bed bug feed exclusively on blood throughout their development. Moreover, they require at least one blood meal to complete each larval stadium and to molt, and multiple blood meals support cycles of egg production^9^. Bed bugs mitigate the nutritional deficiency of blood through a symbiotic association with a Gram-negative intracellular α-proteobacterium, *Wolbachia* (*w*Cle, *Wb*) (subgroup F)^10^. *Wolbachia* species are estimated to be present in 40% to 66% of arthropod species^11,12^ and they impose a wide range of effects on their hosts. *Wolbachia* are widely known for various negative effects on their host, including reproductive manipulation causing feminization, male-killing, parthenogenesis and cytoplasmic incompatibility^13^. *Wolbachia* can also be beneficial to its host. In some *Drosophila* species, *Wolbachia* protects against infection by RNA viruses^14^, and in silkworms it can aid in ovary development^15^. Bed bugs are unique in their use of *Wb* as a nutritional symbiont. Bed bugs house *Wb* in a gonad-associated bacteriome, and *Wb* is found in oocytes, which guarantees its vertical transmission to progeny^16^.

A pivotal approach to investigating microbe-host interactions in symbiotic associations is to produce aposymbiotic host insects by eliminating the endosymbiont. Using rifampicin-supplemented blood to eliminate or reduce the *Wb* titer in *C. lectularius* has been indispensable to understand vitamin provisioning in bed bugs^16–18^. Genomic, nutritional, and physiological studies revealed that *Wb* produces riboflavin and biotin^17^, and its genome has partial pathways for thiamine, folate, and pyridoxine^18^. When *Wb* is eliminated from bed bugs, the host has lower survival, nymphs development is slower, and egg production is affected; however, both development and reproduction improve in aposymbiotic bed bugs fed blood supplemented with B-vitamins^18^. Although these ground-breaking studies clearly confirmed the involvement of *Wb* in provisioning B-vitamins to bed bugs, two aspects of this relationship remain unclear. First, fully eliminating *Wb* with traditional antibiotic treatments is difficult, likely because *Wb* in bed bugs is housed in a specialized bacteriome that may protect it from xenobiotics in the hemolymph. Indeed, in previous studies, only approximately 18% of the rifampicin-treated bed bugs were *Wb*-free (no detectable *Wb* by qPCR), while the rest were *Wb*-deficient, with titers ranging from 10^3^ to 10^6^ *Wb wsp* gene copies per insect; normal bed bugs had 10^3^ to 10^8^ *wsp* gene copies per insect^16^. Second, antibiotics are known to have adverse side effects beyond their intended effects on bacteria^19^. In studies that use antibiotics during physiological and life history experiments, antibiotic residues in tissues and their side-effects may confound results because it is difficult to include appropriate control insects in such a design.

In this study we eliminated *Wb* using the antibiotic rifampicin, reared them for one generation without rifampicin-supplementation, and used rigorous droplet digital PCR for absolute quantification of *Wb* levels in nymphs and adults. We examined development and reproduction in aposymbiotic bed bugs and the effects of B-vitamins in ameliorating the absence of the endosymbionts. This study confirms and extends previous results on the role of *Wb* as a mutualist that provides B-vitamins to its *C. lectularius* host.

## Results

### *Wolbachia* quantification

**Figure 1:**
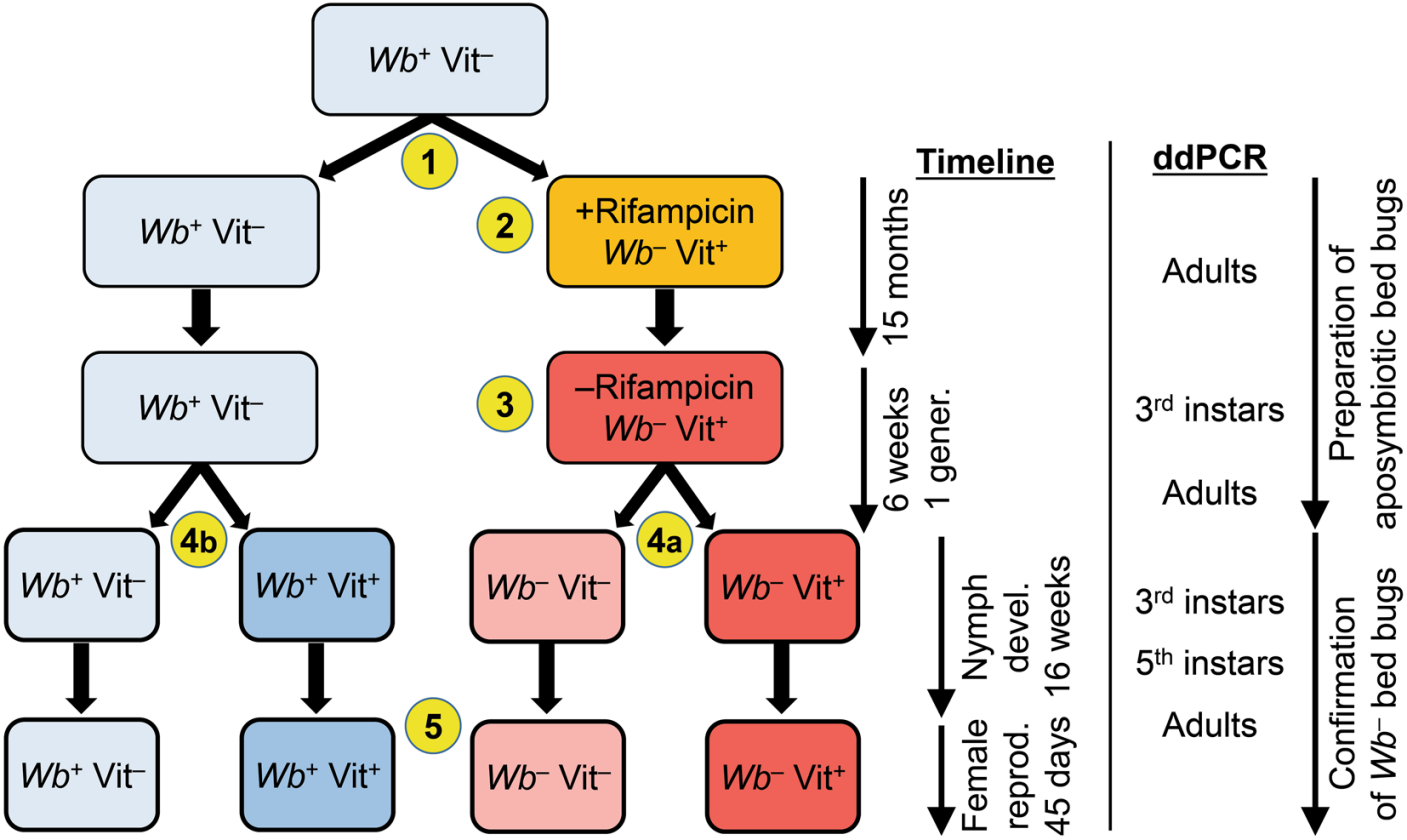
Experimental design. A laboratory colony (*Wolbachia*-present, *Wb*^+^) was fed blood with no added vitamins (Vit^–^). The colony was split into two lines (**1**) *Wb*^+^ and *Wolbachia*-absent (aposymbiotic, *Wb*^–^). The *Wb*^+^Vit^–^ line was fed antibiotic-fortified but not vitamin-supplemented blood for 15 months. The *Wb*^–^Vit^+^ line was generated by concurrently feeding bugs on blood fortified with rifampicin and the K&M mix of B-vitamins for multiple generations for 15 months (**2**). To clear the antibiotic from the colony, *Wb*^–^Vit^+^ bed bugs were reared for 6 weeks (one generation) on rifampicin-free blood supplemented with the B-vitamin mix (**3**). When adults emerged in the *Wb*^–^Vit^+^ treatment group, they were placed in oviposition vials and first instars were collected and split into two treatment groups (**4a**) *Wb*^–^Vit^–^ and *Wb*^–^Vit^+^. The *Wb*^+^Vit^–^ control line was concurrently fed non-supplemented blood, and when adults emerged in the *Wb*^+^Vit^–^ group, they were placed in oviposition vials and first instars were collected and split into control (*Wb*^+^Vit^–^) and vitamin-supplemented (*Wb*^+^Vit^+^) lines (**4b**). Nymphs in the four treatment groups were fed weekly and their development recorded for 16 weeks. As they emerged, adult females and males within the same treatment group were paired (**5**), fed their respective treatment blood every five days for 45 days, and eggs counted every five days. Adults were sampled twice and nymphs were sampled once for ddPCR during the course of generating the *Wb*^–^ line, as indicated. During nymph development of the experimental groups, third instars, fifth instars, and newly emerged adults were sampled from each of five replications for *Wb* quantification by ddPCR.

The experimental design is described in Fig. 1. The *Wb* titers were quantified by ddPCR assay in 125 bed bugs comprising third instars, and adult females during the preparation of an aposymbiotic colony and third instars, fifth instars, adult females, and adult males from four experimental treatments.

**Figure 2:**
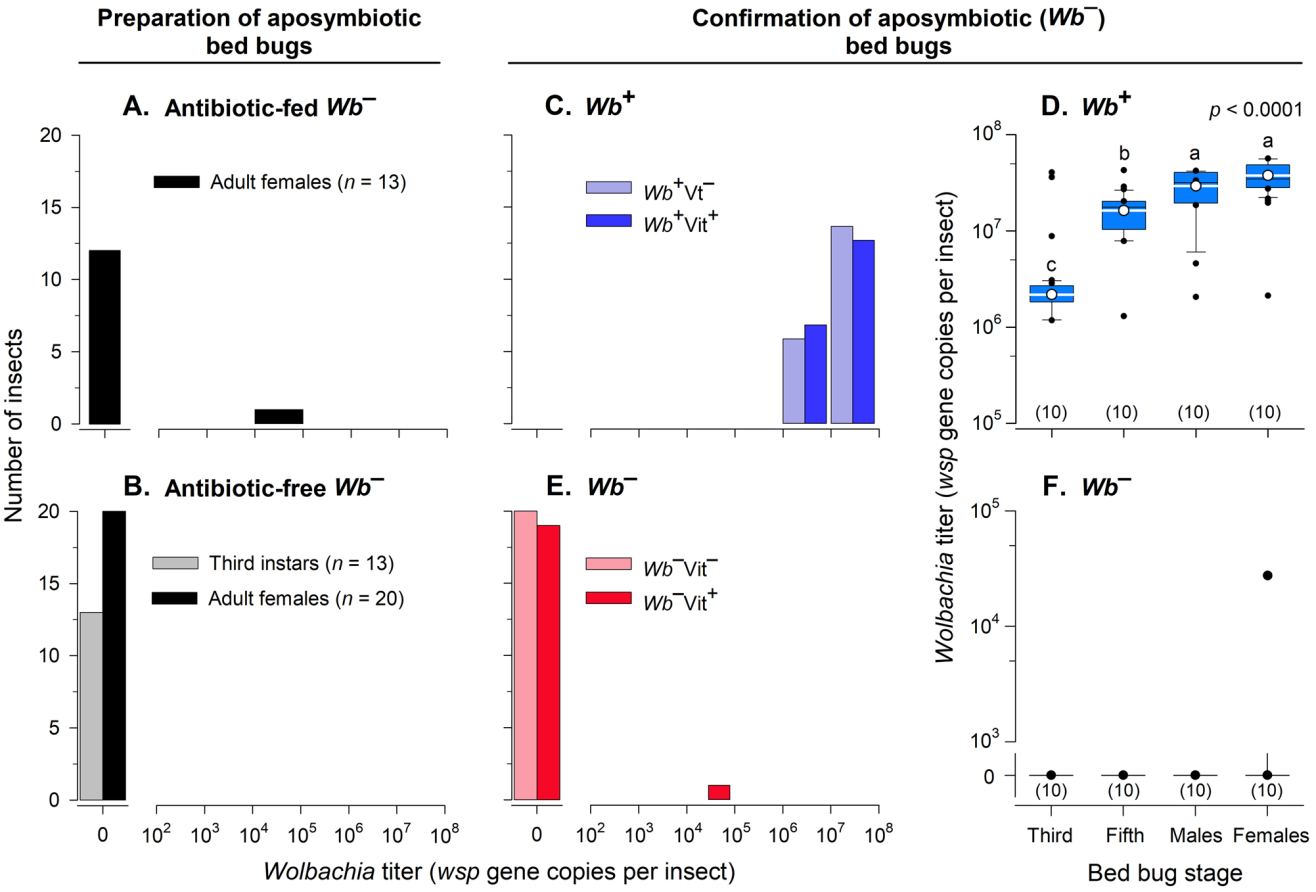
*Wolbachia* quantification of adults and third and fifth instar bed bugs by ddPCR. Twelve out of 13 antibiotic-fed females were *Wb-*free and one female had a significantly lower *Wb* titer (**A**). All 20 females and 13 third instar nymphs in the antibiotic-free generation were *Wb-*free (**B**). All 20 *Wb*^+^ adults, and all 20 *Wb*^+^ third and fifth instars had *Wb* (**C**). Adults had significantly higher *wsp* gene copies than nymphs, and *Wb* titers were significantly higher in fifth than in third instars (**D**). *Wolbachia* was not detected (< 0.5 *wsp* copies per µl) in all 20 *Wb*^–^ third and fifth instars, all 10 *Wb*^–^ adult males, and 9 out of 10 *Wb*^–^ adult females (**E**, **F**).

*Wolbachia* was absent in 12 of 13 females in the antibiotic-fed aposymbiotic preparation colony group. Only one female had a detectable level of *Wb* but the titer was greatly reduced (Fig. 2A). Only the offspring from the 12 *Wb*^–^ females were used in the next antibiotic-free generation. All 13 third instars and 20 females did not have detectable levels of *Wb* in the antibiotic-free *Wb*^–^ bed bugs (Fig. 2B).

Qualitative conventional PCR of the 16S rRNA and *wsp* gene targets of experimental bugs showed high bacteria and *Wb* in *Wb*^+^ samples and low or no bacteria or *Wb* in *Wb*^–^ samples. However, all samples were positive for the bed bug *elf* gene (Supplementary Methods and Fig. S1). In quantitative ddPCR, *Wb* was detected in all 40 *Wb*^+^ samples and we found no significant difference in the *Wb* titer in *Wb*^+^ bed bugs either provisioned with B-vitamins or not (*Wb*^+^Vit^+^ vs. *Wb*^+^Vit^–^) (Fig. 2C). However, the *Wb* concentration was significantly different (*p* < 0.0001) among the four life stages (Fig. 2D). *Wolbachia* titers in the *Wb*^+^ bed bugs were significantly higher in adults than in nymphs (*p* < 0.05) and fifth instars had nearly tenfold more *Wb* than third instars (*p* < 0.05). In contrast, *Wb* was detected in only one adult female of the 40 *Wb*^–^ bed bugs we tested (Fig. 2E,F). However, the *Wb* density in this female was 1000-fold lower than in *Wb*^+^ adult females.

### Nymph development

#### Percentage nymphs fed

**Figure 3:**
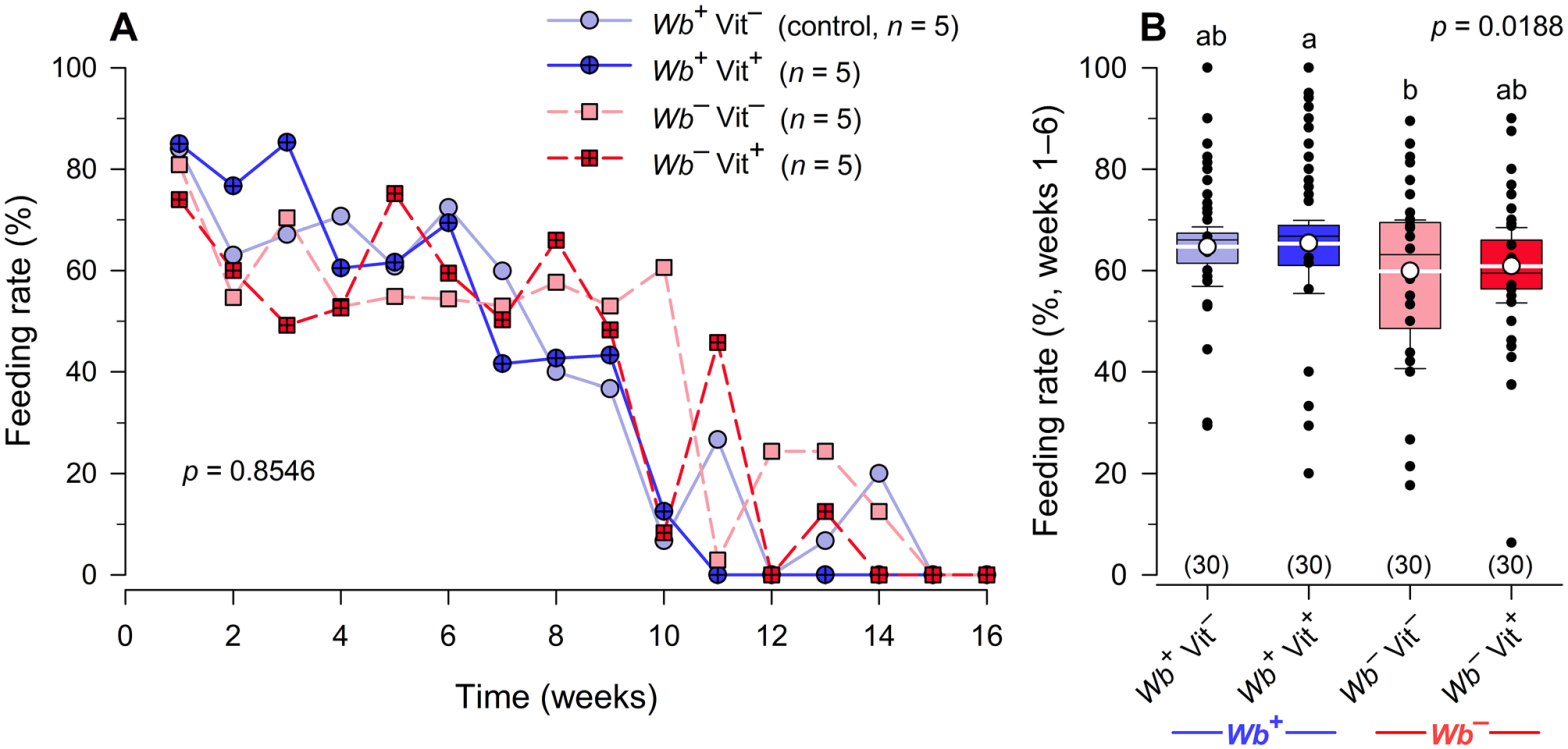
Effects of *Wolbachia* and B-vitamin supplementation on feeding rates of *C. lectularius* nymphs. There were no significant differences among the four treatment groups in percentage nymphs that fed in weekly feeding sessions (Repeated measures ANOVA, *F*_3,9_ = 0.2569, *p* = 0.8546) (**A**), but there was a significant difference among treatments in the percentage that fed each week during weeks 1 to 6 (One-way ANOVA, *F*_3,116_ = 3.4544, *p* = 0.0188) (**B**). Week 6 represents when adults began to emerge in all four treatment groups (Fig. 4A) and the feeding rate is based on the nymphs remaining in the vial. The mean is shown as a white circle and white horizontal line and sample size is indicated in parentheses. Percent data were arcsine square root transformed prior to analysis. Groups that do not share letters are significantly different from each other (Tukey’s honestly significant difference test, *p* < 0.05).

The weekly percentage nymphs that fed was variable over time, but not consistently different among the four treatments over 16 weeks (*F*_3,9_ = 0.2569, *p* = 0.8546). The percentage nymphs that fed decreased in all treatments between weeks 6 and 16 (Fig. 3A). More nymphs fed in the *Wb*^+^Vit^+^ treatment than in the *Wb*^–^Vit^–^ treatment in weeks 1–6 (*F*_3,116_ = 3.4544 *p* = 0.0188) (Fig. 3B).

#### Adult emergence and nymph survivorship

**Figure 4:**
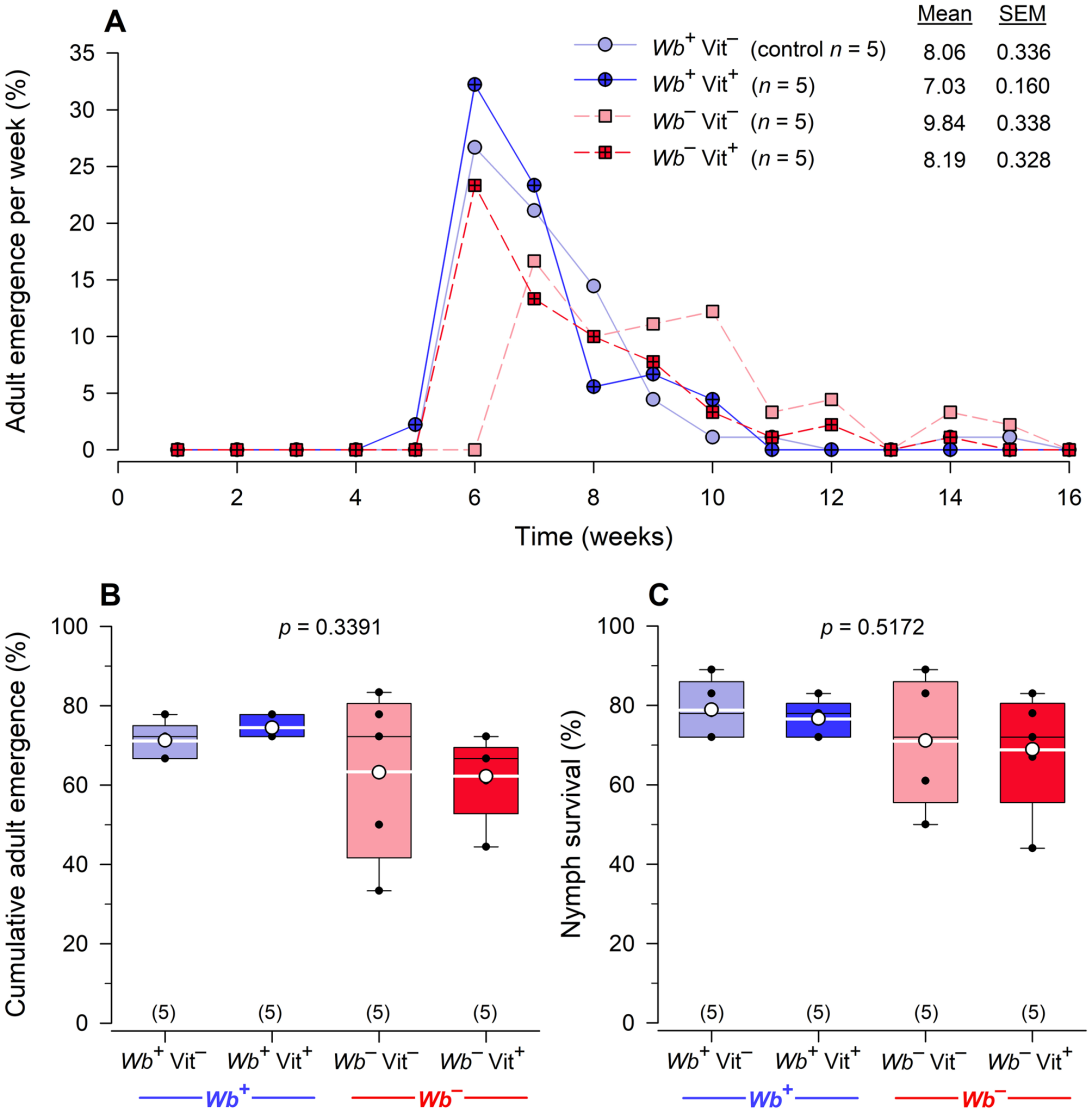
Effects of *Wolbachia* and B-vitamin supplementation on *C. lectularius* nymph development and adult emergence over 4 months. Mean adult emergence time was significantly longer in the *Wb*^*–*^Vit^–^ group (9.84 weeks) (Product-limit survival analysis: Wilcoxon *X*^2^ = 46.6316, df = 3, *p* < 0.0001) (**A**). There were no significant differences among the four treatments in the average cumulative percentage of adults that emerged in each treatment after 16 weeks (One-way ANOVA, *F*_3,16_ = 1.2070, *p* = 0.3391) (**B**). There were also no significant differences among the four treatments in the average percentage of nymphs that survived the 16-week experiment (One-way ANOVA, *F*_3,16_ = 0.7896, *p* = 0.5172) (**C**). Adults that emerged were included as having survived development. For each box plot, the mean is shown as a white circle and a white horizontal line and sample size is indicated in parentheses.

Mean adult emergence times were shortest for the two *Wb*^+^ treatments (*Wb*^+^Vit^–^ = 8.06 weeks, *Wb*^+^Vit^+^ = 7.03 weeks) and 2 males emerged 1 week earlier than in all other treatments (Fig. 4A). Adult emergence was completed in the *Wb*^+^Vit^+^ treatment by week 10, whereas in the *Wb*^+^Vit^–^ control 3 (4.69%) additional adults emerged between weeks 11–16. In contrast, mean adult emergence times were longest for *Wb*^–^Vit^–^ (9.84 weeks) and adults did not start to emerge until week 6, which was one week after all other treatments. In addition, the *Wb*^–^Vit^–^ treatment continued to emerge every week until the assay was terminated. Therefore, nymphal development was significantly longer for *Wb*^–^Vit^–^ bed bugs than in the other treatments (*W* = 46.6316, df = 3, *p* < 0.0001). Supplementation of blood with B-vitamins caused *Wb*^–^ nymphs to recover normal development with a mean emergence time of 8.19 weeks; both *Wb*^+^ treatments, and the *Wb*^–^Vit^+^ treatment had similar emergence distributions. In the *Wb*^–^Vit^+^ treatment, four adults (7.02%) emerged between weeks 11–16, similar to the *Wb*^+^Vit^–^ control.

By the end of the nymph development assay, on week 16, the total number of adults that emerged from each treatment was unaffected by the absence of *Wb* or the addition of vitamins (*F*_3,16_ = 1.2070, *p* = 0.3391), although there was much greater variation among the five *Wb*^–^Vit^–^ replicates than in the other three treatments (Fig. 4B). Nymph survival was also unaffected by the absence of *Wb* or B-vitamin supplementation of the blood meal (*F*_3,16_ = 0.7896, *p* = 0.5172) (Fig. 4C).

### Adult body size, fecundity, hatch rate, and survival

#### Adult body size

**Figure 5:**
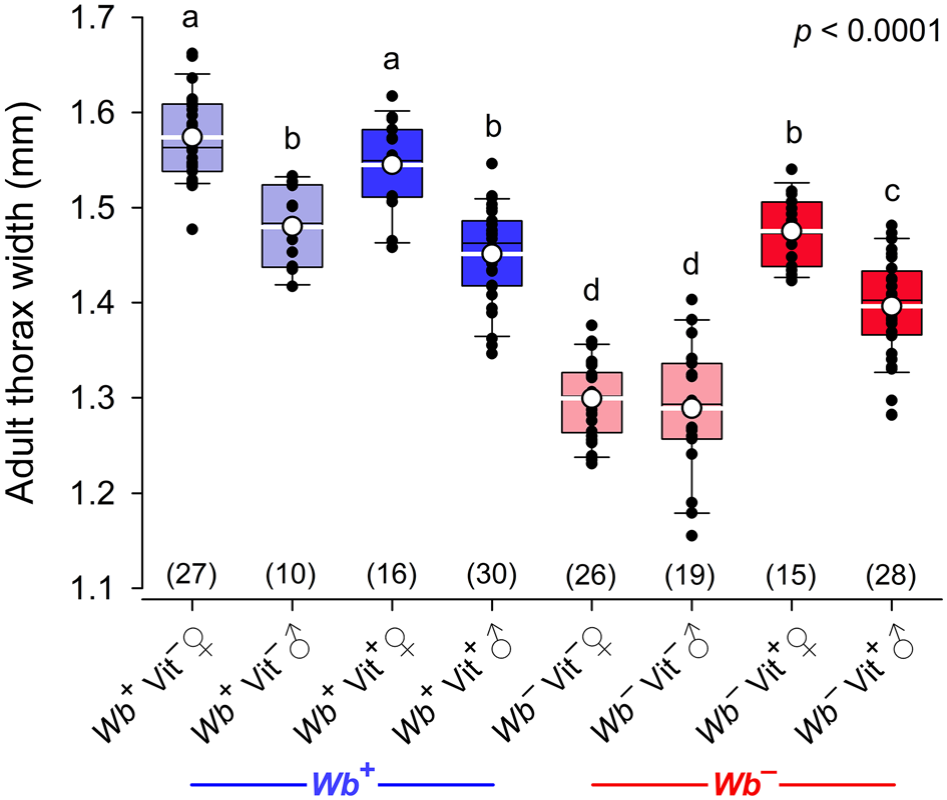
Effects of *Wolbachia* and B-vitamin supplementation on thorax width of female and male *C. lectularius* adults. *Wb*^*–*^Vit^–^ females and males were smaller than in all other treatments (2-way ANOVA, *F*_7,163_ = 102.954, *p* < 0.0001). Although vitamin supplementation (*Wb*^–^Vit^+^) significantly increased thorax width in both sexes compared with *Wb*^–^Vit^–^ bed bugs, the vitamin-fortified blood was not sufficient to recover thorax width to the level of control bed bugs (*Wb*^+^Vit^–^). The mean is shown as a white circle and a white horizontal line and sample size is indicated in parentheses. Groups that do not share letters are significantly different from each other (Tukey’s honestly significant difference test, *p* < 0.05).

Thorax width in both sexes of the *Wb*^–^Vit^–^ bed bugs was significantly smaller than in the other three treatments (2-way ANOVA, *F*_7,163_ = 102.954, *p* < 0.0001) (Fig. 5). Blood fortified with B-vitamins was not sufficient to fully recover the thorax width to that of *Wb*^+^ bed bugs when *Wb* was absent, because both sexes of *Wb*^–^Vit^+^ bed bugs were still significantly smaller than the respective *Wb*^+^ bed bugs. In normal *C. lectularius*, adult females are larger than males, and thorax width of females in the *Wb*^+^Vit^–^ control was 0.0943 mm (6.4%) larger than in males (*t*-test, *t* = 5.7639, *p* < 0.0001). Likewise, thorax width of females in the *Wb*^+^Vit^+^ and *Wb*^–^Vit^+^ treatment groups was 0.0936 mm (6.5%) and 0.0791 mm (5.7%) larger, respectively, than males, and these differences were statistically significant in both (*Wb*^+^Vit^+^, *t* = 6.2502, *p* < 0.0001; *Wb*^–^Vit^+^, *t* = 5.2895, *p* < 0.0001). In contrast, thorax width of *Wb*^–^Vit^–^ females was only 0.0107 mm (0.8%) wider than in males (*t* = 0.6622, *p* = 0.5114) (Fig. 5). These results indicate that the absence of *Wb* affects female development and ultimately adult female size more than it does in males. Moreover, it appears that B-vitamin supplementation can partially, but not completely, restore female development and the size differential between females and males.

#### Fecundity

**Figure 6:**
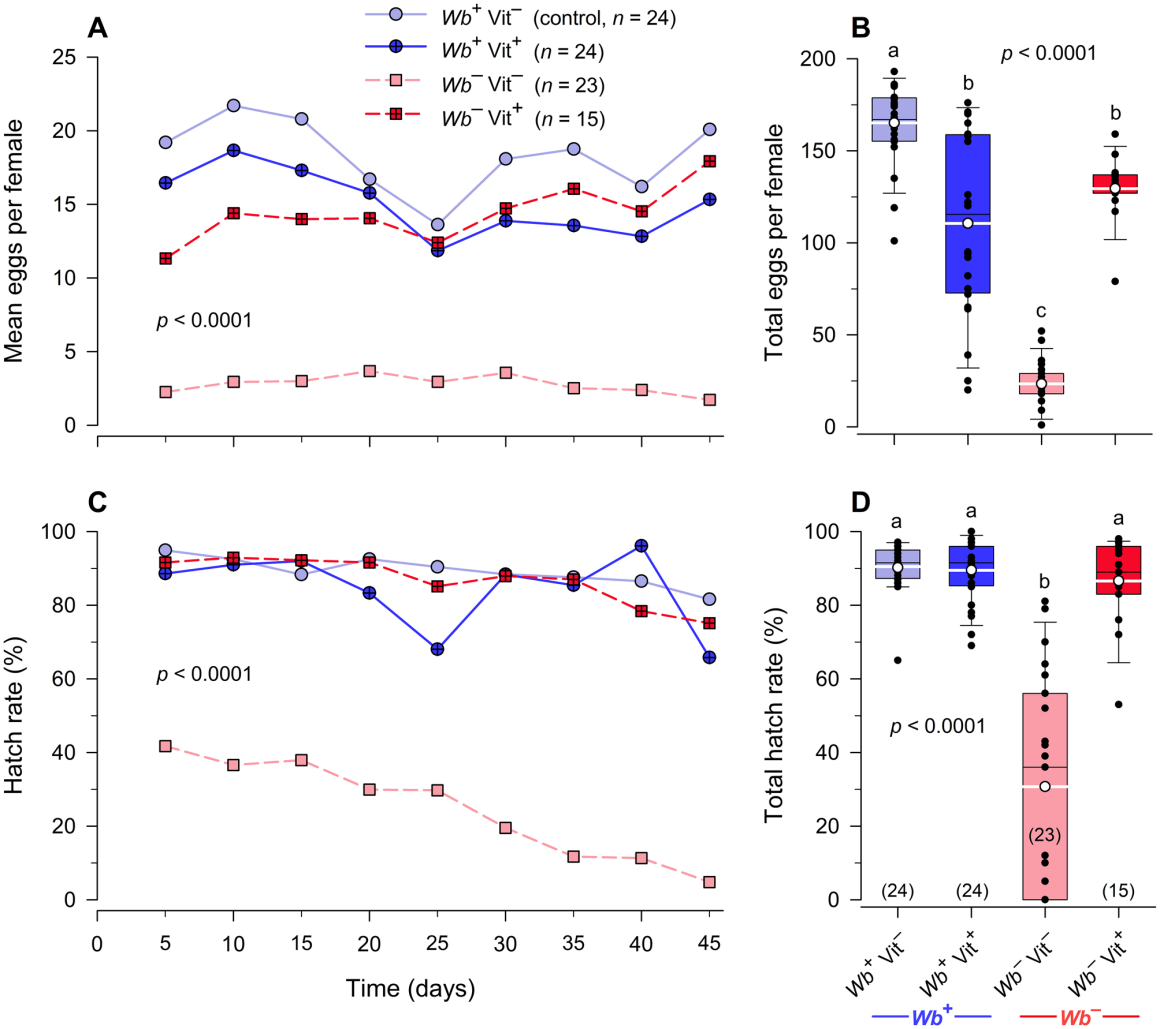
Effects of *Wolbachia* and B-vitamin supplementation on egg production and egg hatch in *C. lectularius*. Average egg production per female per week (**A**), total egg production per female (**B**), average egg hatch per female per week (**C**), and total egg hatch (**D**) during the 45-day experiment. In (**A**) and (**C**), each time point reflects the average number of eggs laid or percent hatch during the preceding 5-day interval. *Wb*^–^Vit^–^ females laid consistently fewer eggs over time (Repeated measures ANOVA, *F*_3,66_ = 188.277, *p* < 0.0001), and laid fewer total eggs (One-way ANOVA, *F*_3,82_ = 90.917, *p* < 0.0001) than females in all other treatments. *Wb*^–^Vit^–^ females also had consistently lower egg hatch over time (Repeated measures ANOVA, *F*_3,66_ = 56.2279, *p* < 0.0001), and lower total percent hatch (One-way ANOVA, *F*_3,82_ = 55.5504, *p* < 0.0001) than females in all other treatments. Percent hatch data were arcsine square root transformed prior to analysis. Groups that do not share letters are significantly different from each other (Tukey’s honestly significant difference test, *p* < 0.05). Summary descriptive statistics (mean, SD, SEM, *n*) for (**A**, **C**) are available in Supplementary Information.

*Wb*^–^Vit^–^ females laid consistently fewer eggs at every 5-day interval (Fig. 6A) and fewer total eggs per female than *Wb*^+^Vit^–^ females during the 45-day experiment. Oviposition in B-vitamin fed aposymbiotic females (*Wb*^–^Vit^+^) increased over time, from 11.3 eggs on days 0–5 to 17.9 eggs on days 41–45 (58.2% increase) reaching within 10.7% (2.15 eggs) of the oviposition rate of normal control females. The latter females changed minimally from 19.2 eggs on day 5 to 20.1 eggs on day 45 (4.6% increase) (Fig. 6A). Each control female (*Wb*^+^Vit^–^) oviposited 165.2 ± 6.2 (SEM) eggs over 45 days, whereas the *Wb*^–^Vit^–^ females oviposited only 23.4 ± 6.3 eggs per female. The addition of B-vitamins to *Wb*^–^ females (*Wb*^–^Vit^+^) significantly increased the number of eggs laid per female to 129.5 ± 7.8 (453% increase) but did not fully recover oviposition to control levels (ANOVA, Tukey’s HSD, *F*_3,82_ = 90.9167, *p* < 0.0001) (Fig. 6B). Unexpectedly, the *Wb*^+^Vit^+^ females oviposited fewer eggs than the control females (*Wb*^+^Vit^–^), which appears to indicate a negative effect of B-vitamins supplementation in *Wb*^+^ females (Fig. 6B).

#### Percentage egg hatch

The hatch rate was consistently high in the *Wb*^+^Vit^–^ and *Wb*^+^Vit^+^ treatments during the 45-day experiment (Fig. 6C) with mean hatch percentages of 90.5 ± 1.4% and 89.5 ± 1.8%, respectively, which did not differ statistically (ANOVA, Tukey’s HSD) (Fig. 6D). In contrast, egg hatch rate in *Wb*^–^Vit^–^ females declined by 88.5%, from 41.7% on day 5 to only 4.8% on day 45, while in normal control females it declined only 14.0% from 94.9% to 81.6% during the same interval (ANOVA, *F*_3,66_ = 56.2279, *p* < 0.0001). B-vitamins fully recovered the effect of *Wb* elimination even on day 5 (91.6% egg hatch) (Fig. 6C). Egg hatch rate over the 45-day interval in the *Wb*^–^Vit^–^ treatment averaged 30.7 ± 3.6%, significantly lower than in the two *Wb*^+^ treatments (ANOVA, Tukey’s HSD, *F*_3,82_ = 55.5504, *p* < 0.0001). However, the percent hatch per female in *Wb*^–^ females was fully recovered to control levels (*Wb*^–^Vit^+^ = 86.6 ± 3.1%) by adding B-vitamins to their blood meals (Fig. 6D).

#### Female survival

**Figure 7:**
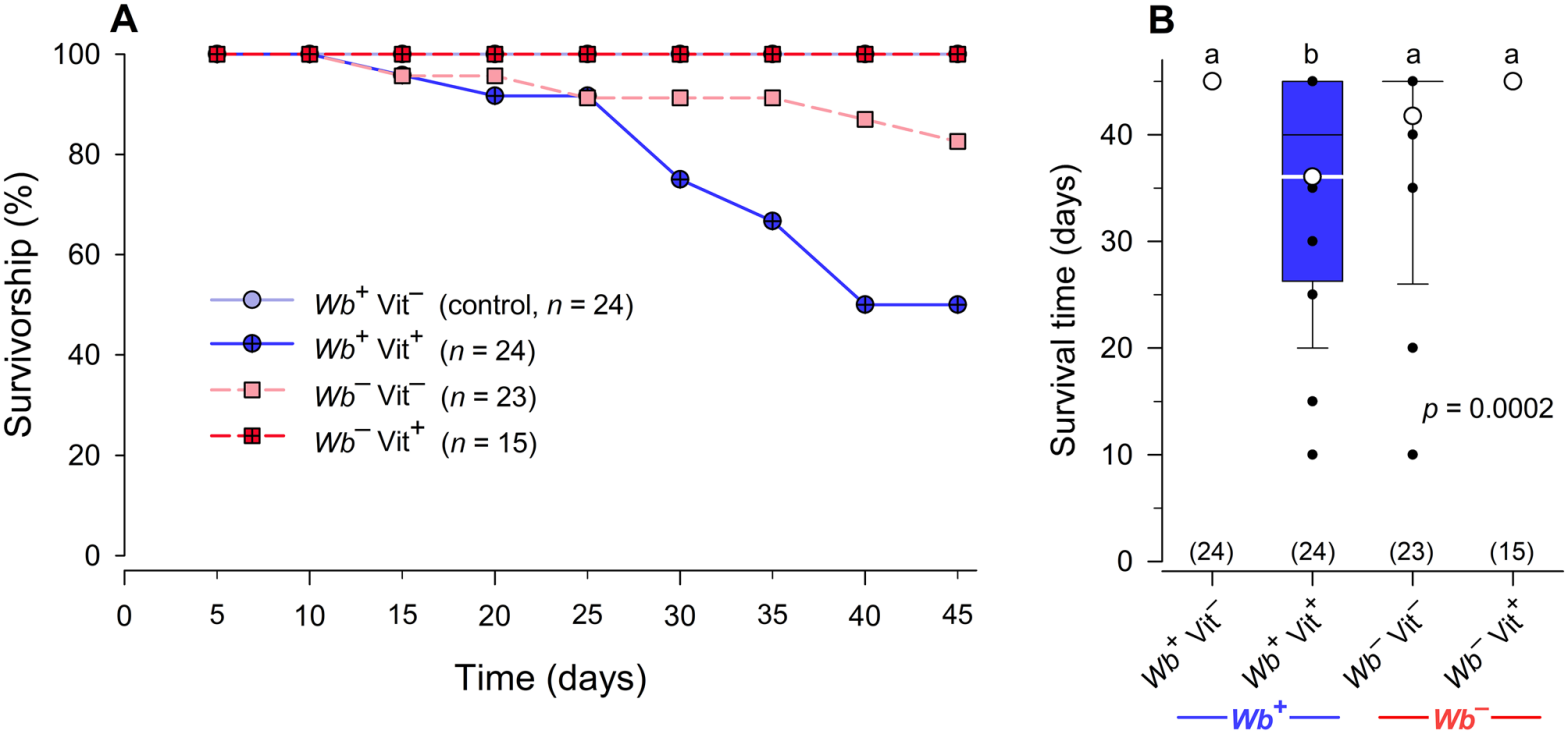
Effects of *Wolbachia* and B-vitamin supplementation on 45-day survivorship of female *C. lectularius* used in the fecundity assay. The *Wb*^+^Vit^–^ line is obscured by the *Wb*^+^Vit^+^ line, both at 100%. *Wb*^+^Vit^+^ and *Wb*^–^Vit^+^ females experienced 50% and 17.3% mortality, respectively (Product-limit survival analysis: Wilcoxon *X*^2^ = 25.6657, df = 3 *p* < 0.0001) (**A**). The average survival age within the 45-day experiment shows that all females in the *Wb*^+^Vit^–^ and *Wb*^–^Vit^+^ groups survived for 45 days but survival was significantly lower for the *Wb*^+^Vit^+^ (One-way ANOVA, Tukey’s honestly significant difference test, *F*_3,81_ = 7.4441, *p* = 0.0002) (**B**).

Mean percent survival of adult females in the control treatment (*Wb*^+^Vit^–^) was 100% during the 45-day experiment. Female survival in the *Wb*^–^Vit^–^ treatment was intermediate after 45 days (82.6%), but the addition of B-vitamins to *Wb*^–^ females fully recovered their survival to 100%. (*X*^2^ = 25.6657, df = 3, *p* < 0.0001) (Fig. 7A). As with egg production, the addition of vitamins to *Wb*^+^ females (*Wb*^+^Vit^+^) significantly decreased their survival to 50.0% after 45 days (ANOVA, Tukey’s HSD, *F*_3,81_ = 7.4441, *p* = 0.0002) (Fig. 7B), again suggesting a negative effect of B-vitamins supplementation in *Wb*^+^ females.

### Comparison of two B-vitamin mixes

**Figure 8:**
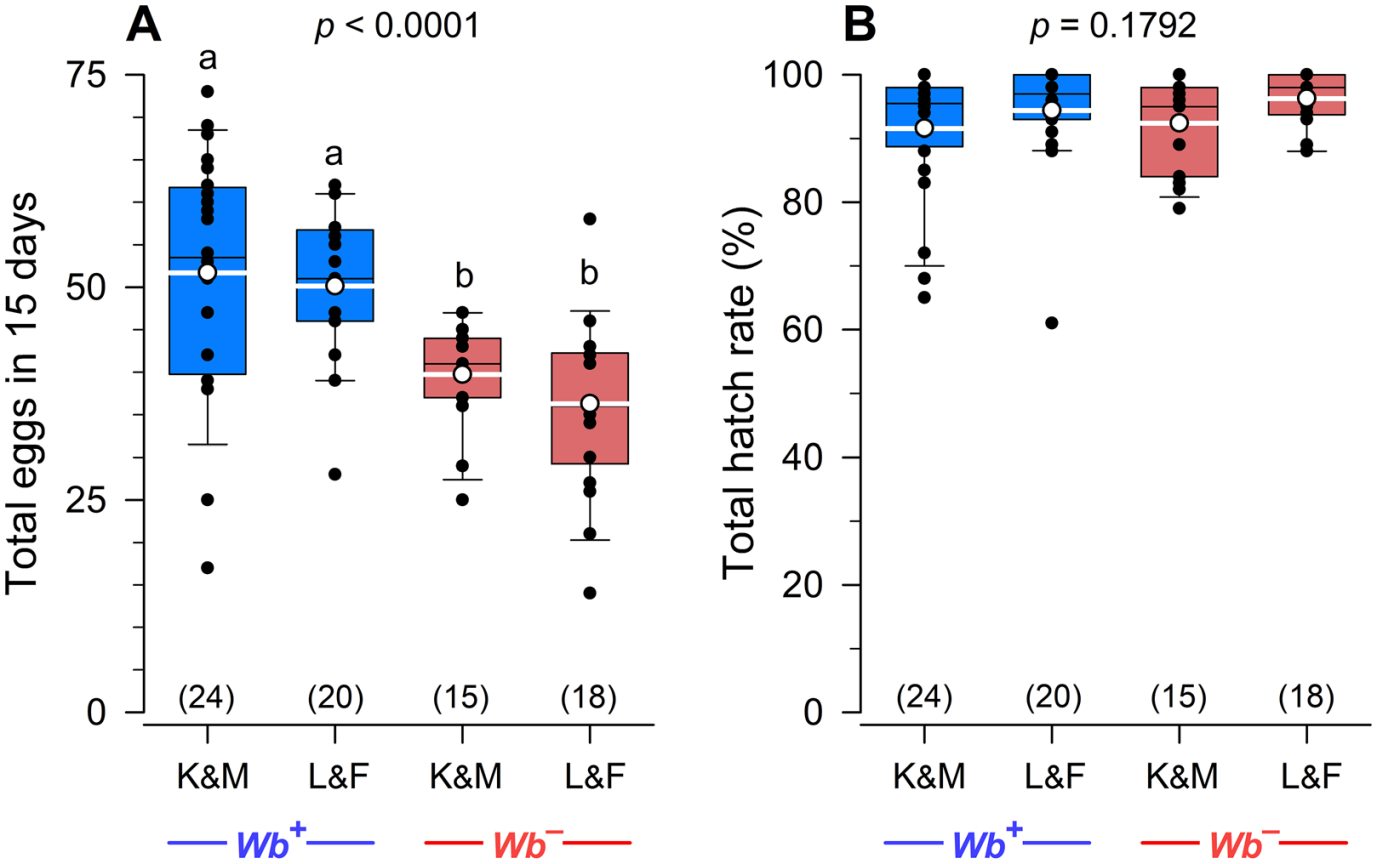
Effects of *Wolbachia* and two B-vitamin supplements on egg production and egg hatch in *C. lectularius*. *Wb*^–^ and *Wb*^+^ bed bugs were fed blood supplemented with the Kao and Michayluk vitamin mix (K&M) or the Lake and Friend vitamin mix (L&F). (**A**) Females in both *Wb*^*–*^ treatments laid fewer eggs than in the *Wb*^+^ treatments, independent of the type of vitamin mix (One-way ANOVA, *F*_3,73_ = 9.9333, *p* < 0.0001). (**B**) There were no significant differences among the four treatment groups in percent hatch (One-way ANOVA, *F*_3,73_ = 1.6778, *p* = 0.1792). Percent hatch data were arcsine square root transformed prior to analysis. Groups that do not share letters are significantly different from each other (Tukey’s honestly significant difference test, *p* < 0.05).

Because we used a different B-vitamin mix (K&M) than previous researchers (L&F)^16–18^, we directly compared the two mixes in adult females. We did not include a *Wb*^+^Vit^–^ treatment because only the vitamin mixes were being compared. Figure 8A shows that *Wb*^+^ females provided either of the two vitamin mixes produced similar numbers of eggs. Likewise, *Wb*^–^ females provided either of the two vitamin mixes produced similar numbers of eggs. However, the *Wb*^–^ females supplemented with either of the two vitamin mixes produced significantly fewer eggs than similarly treated *Wb*^+^ females (ANOVA, Tukey’s HSD, *F*_3,73_ = 9.9333, *p* < 0.0001). Egg hatch was high (range: 91.6 to 96.3%) across all four treatments (ANOVA, Tukey’s HSD, *F*_3,73_ = 1.6778, *p* = 0.1792) (Fig. 8B), consistent with our previous results (Fig. 6D).

## Discussion

Our results highlight the importance of *Wolbachia* as a nutritional mutualist in bed bugs. The elimination of *Wb* significantly increased nymph development time and reduced adult size and survivorship, eggs laid per female, and hatch rate. When aposymbiotic bed bugs were fed blood supplemented with B-vitamins, nymph development time, egg hatch rate and adult survival were fully recovered to the levels of control bed bugs, but adult size and per capita egg production only partially recovered. This is the first study in bed bugs that fully eliminated *Wb* before and throughout experimentation, and that mitigated the side-effects of antibiotics by discontinuing the use of antibiotics for a generation before the start of experiments. It is also the first study that used a complete design that included normal *Wb*-present (*Wb*^+^) control bed bugs in all fitness-related assays. Our results support and extend the findings of previous studies that used a range of *Wb*-free (*Wb*^–^) and *Wb*-deficient bed bugs. Our results also add life history traits that are affected by *Wb* to previously documented traits.

### What motivated this work?

Pioneering studies of the *Wb-*bed bug symbiosis confirmed that *Wb* is housed in a gonad-associated bacteriome and is vertically transmitted, and demonstrated that *Wb* produces biotin and riboflavin that benefit the bed bug host^16–18^. Hosokawa et al.^16^ examined several fitness-related traits, including the rates of egg and nymph development, and the percentage of nymphs that emerged into adults. Their results showed that *Wb* was an essential nutritional symbiont for normal growth and reproduction of *C. lectularius*. Four main reasons motivated us to re-examine these traits and consider additional traits. First, in the previous studies, “aposymbiotic” bed bugs obtained with rifampicin treatments were shown to be *Wb*-deficient, with titers reduced by 100 to 10,000 *wsp* gene copies; only 8 of 30 (26.7%) of the “aposymbiotic” bed bugs were shown by qPCR to be free of *Wb*^16^. Second, in previous work, rifampicin was used throughout the experiments in two treatment arms—“aposymbiotic” bed bugs fed blood and “aposymbiotic” bugs fed blood supplemented with B-vitamins^16^. Adverse effects of the antibiotic might therefore affect life-history traits throughout the experimental period. Third, in some experiments, control bed bugs harboring *Wb* were not used^17,18^, so it was difficult to determine if B-vitamins fully or partially recovered the traits that were examined. Finally, we felt that some incongruent results for numbers of eggs laid and adult emergence in Hosokawa et al.^16^ and Moriyama et al.^18^ required closer examination. For example, the number of eggs laid was unaffected by antibiotic treatment in the early study^16^, but the same treatment resulted in complete elimination of egg production in the later study^18^. Likewise, adult emergence was greatly reduced in the 2010 and 2014 reports^16,17^, but the 2015 study^18^ showed almost no adult emergence in the *Wb*-deficient colonies. It is important to emphasize, however, that although our results differ quantitatively from these previous studies, they broadly support the general conclusions of these groundbreaking studies.

To address these four issues, we developed procedures for absolute quantification of *Wb* titers in bed bugs and used these procedures to confirm that our rifampicin-treated bed bugs were indeed aposymbyotic. Compared to qPCR, droplet digital PCR (ddPCR) has higher sensitivity, specificity, and reproducibility; it can quantify low target numbers^20^ (our empirically derived lower detection level was 0.5 gene copies per µl) and it provides the absolute quantification of DNA in a sample^21,22^, making it an appropriate technology for confirming the absence of *Wb* in bed bugs. Using ddPCR, we showed that 100% of the third instars, fifth instars, and adult males, and 90% (9 of 10) of the adult females tested in our experiments were *Wb-*free; the one adult female that contained *Wb* was highly *Wb-*deficient with 1000-fold fewer *Wb wsp* gene copies than normal *Wb*^+^ females. These procedures ensured that the changes in life history traits attributed to the presence or absence of *Wb* and B-vitamin supplementation were not confounded by incomplete elimination of the endosymbiont.

Persistent antibiotic treatments have many unintended side effects. Yet, many studies on insect-microbe symbiosis administer large doses of antibiotics during the implementation of experiments. If available, chemicals with similar side effects but no antibiotic properties could be used in control treatments. However, this approach is complicated and we are not aware of its use in research on insect symbiosis. Instead, we treated a colony of bed bugs with rifampicin for multiple generations (experimental design in Fig. 1), confirmed that they were aposymbiotic by ddPCR, then reared one generation without rifampicin to clear the antibiotic from the colony, and again confirmed by ddPCR that they remained aposymbiotic throughout the experiments. We also included *Wb*^+^ control groups in all fitness tests to determine if vitamins fully or partially recovered the effect of *Wb* elimination.

### Specific effects of aposymbiosis on bed bugs and B-vitamin supplementation

Table 1 summarizes the biological traits tested in our study, whether the elimination of *Wb* affected the trait and if blood meals fortified with B-vitamins could recover the trait to the levels observed in control bed bugs (*Wb*^+^Vit^–^).

**Table 1:** Summary of the effects of *Wolbachia* (*Wb*) on life-history traits of *C. lectularius* and whether the addition of B-vitamins to blood meals partially or fully restored the trait to the level of control (*Wolbachia*-present, *Wb*^+^) insects.

Life-history trait	Affected by *Wb*? (*Wb*^–^Vit^–^ vs. *Wb*^+^Vit^–^)	Does B-vitamin supplementation mitigate the deficit? Does it fully or partially recover the deficit? (*Wb*^–^Vit^–^ vs. *Wb*^–^Vit^+^)
***Percentage nymphs that fed (Fig. ******3******)***	***No, Wb***^***–***^***Vit***^***–***^*** = Wb***^***+***^***Vit***^***–***^	**N/A**
**Rate of nymphal development (Fig. ****4****)**	**Yes, *Wb***^**–**^**Vit**^**–**^** < *Wb***^**+**^**Vit**^**–**^	**Yes, fully**
***Number of first instars that became adults (Fig. ******4******)***	***No, Wb***^***–***^***Vit***^***–***^*** = Wb***^***+***^***Vit***^***–***^	**N/A**
*Adult thorax width (Fig. **5**)*	*Yes, Wb*^*–*^*Vit*^*–*^* < Wb*^*+*^*Vit*^*–*^	*Yes, partially*
*Number of eggs per female (Fig. **6**)*	*Yes, Wb*^*–*^*Vit*^*–*^* < Wb*^*+*^*Vit*^*–*^	*Yes, partially*
**Percent egg hatch (Fig. ****6****)**	**Yes, *Wb***^**–**^**Vit**^**–**^** < *Wb***^**+**^**Vit**^**–**^	**Yes, fully**
**Female survivorship (Fig. ****7****)**	**Yes, *Wb***^**–**^**Vit**^**–**^** < *Wb***^**+**^**Vit**^**–**^	**Yes, fully**

Bold italics indicates no effect of *Wb* removal on the fitness-related traits, italics indicates traits that were partially recovered by the addition of B-vitamins, and bold indicates fitness-related traits that were fully recovered to control levels with the addition of B-vitamins.

Our results showed that the elimination of *Wb* did not affect feeding rate in bed bugs. It also did not affect survival of nymphs and the cumulative emergence of adults. The latter results were notably different from previous findings^16–18^. Consistent with earlier findings^16^, however, development of aposymbiotic nymphs was slower and their mean time to adult emergence was 1.8 weeks (22.2%) longer than control (*Wb*^+^Vit^–^) nymphs. Supplementation of the blood meal with B-vitamins restored the rate of development of aposymbiotic nymphs and their mean time to adult emergence was only 0.14 weeks (1.7%) slower than the control nymphs.

Adult size was significantly lower in aposymbiotic females and males than in normal *Wb*^+^ bed bugs. Interestingly, whereas normal females are significantly larger than normal males, aposymbiotic adults converged on the same smaller size, indicating that the absence of *Wb* affected female size more than male size. Smaller adult size was also observed in aposymbiotic stinkbugs, *Megacopta punctatissma* and *Megacopta cribraria*^23^, but the underlying mechanisms remain unknown. In our study, B-vitamin supplementation recovered the sexual dimorphism in adult size, but body size only partially recovered relative to normal adults. These findings suggest that the recovery may require multiple generations or that *Wb* provisions the bed bug with other factors, including other nutrients, in addition to B-vitamins.

Our results demonstrated dramatic effects of *Wb* on reproduction. Aposymbiotic females oviposited 85.8% fewer eggs than control females (*Wb*^+^Vit^–^), despite being fed every 5 days and remated once. Supplementation of the blood meal with B-vitamins significantly increased per capita oviposition by 5.5-fold, but it was only partially recovered to the oviposition rate of normal females. Of interest, however, was the observation that in B-vitamins-fed aposymbiotic females (*Wb*^–^Vit^+^) oviposition increased over time, reaching within 10.7% (2.15 eggs) of the oviposition rate of normal control females on days 41–45, but control females changed minimally. These observations suggest that the recovery of egg production with B-vitamin supplementation is a gradual process, and it may require more than 45 days for their full recovery.

In contrast, egg hatch rate in aposymbiotic females declined by 88.5% between days 5 and 45, whereas in normal control females it declined only 14.0% during the same period. B-vitamins fully recovered the effect of *Wb* elimination as early as day 5 (91.6% hatch), and the hatch rate of *Wb*^–^Vit^+^ eggs declined only 18% between days 5 and 45, similar to control females. These results are generally consistent with previous findings of low egg development in aposymbiotic *C. lectularius*^16^.

### Effects of adding B-vitamins to Wb^+^ bed bugs

Bed bugs require a blood meal before every molt^9^ and have 5 instars. Therefore, we did not expect adults until week 6, and indeed 37.5% of the adult emergence in the *Wb*^+^Vit^–^ control treatment occurred on week 6. The addition of B-vitamins to normal (*Wb*^+^) bed bugs sped up adult emergence, and the mean adult emergence of vitamin-supplemented *Wb*^+^Vit^+^ nymphs was a week earlier than that of normal bed bugs fed non-supplemented blood; two males (of 67 total, 3%) emerged after only 5 weeks. B-vitamin supplementation of normal bed bugs has not been reported previously, so this is the first report of faster nymph development on B-vitamin supplemented blood.

B-vitamin supplementation of normal nymphs did not affect their cumulative emergence to adults, nymph survival during the 4-month experiment, or adult size. However, it is interesting that the addition of B-vitamins to the blood meals of normal adult females reduced their per capita egg production and female survivorship over 45 days, but not the egg hatch rate. This was unexpected because B-vitamins are water soluble, animals excrete high levels of excess vitamins, and they store only moderate levels of B-vitamins^8^. It is possible that bed bugs are more susceptible to high levels of certain B-vitamins. For example, biotin (B-7), which is essential for processing of lipids, carbohydrates, and amino acids, is only required in minute doses for insects^24^ and dietary overdose of biotin has been shown to be an effective chemosterilant in the house fly, *Musca domestica*^25^ and it reduced fertility in the hide beetle, *Dermestes maculatus*, by suppressing embryogenesis^26^. Because *Wb* is known to produce biotin in bed bugs^17^ it is possible that the low concentration of biotin (10 ng/mL blood) fed to *Wb*^+^ bed bugs similarly affected female fecundity. The mechanisms underlying the differential effects of B-vitamin supplementation on development and reproduction are unknown and should be investigated.

### Differences among studies and limitations of this study

In addition to differences in the *Wb* titers of aposymbiotic bed bugs in this and previous studies, other differences are notable. The bed bug strain used in previous studies^16–18^ to test the biological effects of *Wb* was the Japan Environmental Sanitation Center (JESC) from Kanagawa, Japan, which lacks a facultative γ-proteobacterial symbiont^16–18^. This symbiont is present in the Harold Harlan strain used in the current study, but we did not test whether it was eliminated by the rifampicin treatment. It is plausible that the γ-proteobacterial symbiont is also a nutritional mutualist and it too was eliminated by our extensive rifampicin treatments. This might explain the only partial recovery of several traits with B-vitamin supplementation. It is also important to note that in all studies using antibiotic treatments, the gut bacterial communities were likely dramatically affected or eliminated. Thus, any life history traits that could not be fully recovered in *Wb*-free bed bugs with B-vitamin supplementation might have been affected by a disrupted gut microbiome.

Moreover, as mentioned before, Nikoh et al.^17^ and Moriyama et al.^18^ reared one generation of rifampicin-treated bed bugs without blood meal supplementation with B-vitamins, to eliminate transgenerational carryover of B-vitamins. Insects do not have a large capacity to store B-vitamins^8^, but it is conceivable that this experimental design depleted certain B-vitamins in the developing nymphs. It is unclear if this difference in design contributed to the differences between our studies.

Finally, we used the Kao and Michayluk B-vitamin mix, as did Fisher et al.^27,28^, which differs substantially from previous studies with aposymbiotic bed bugs^16–18^. Nevertheless, direct comparisons of these vitamin mixes showed that both had no effect on egg production in *Wb*-present (*Wb*^+^) bed bugs and both similarly, but only partially, restored egg production in *Wb*-free (*Wb*^–^) females. Both vitamin mixes equally restored hatch rate of *Wb*^–^ females to the high levels of *Wb*^+^ females.

### Outstanding questions on the role of Wolbachia in bed bugs

There are numerous questions to be addressed about the nutritional fitness-related benefits of *Wb* in bed bugs. B-vitamins are co-enzymes in various metabolic reactions^8^, so it is not surprising that *Wb* would contribute to nymph growth and development, egg production and hatch rate. Our study shows the importance of B-vitamins in bed bug development and reproduction, but adult size and egg production results suggest that either our B-vitamin mix was not optimal or that *Wb*, or other symbionts that were eliminated by rifampicin, contribute nutrients to its *C. lectularius* host. Our observations also suggest that B-vitamins aid in the digestion of blood meals. We observed that aposymbiotic bed bugs that were not supplemented with B-vitamins retained blood in their gut for a longer duration than in all other treatments. B-vitamin supplementation in aposymbiotic bed bugs partially recovered faster blood meal digestion. In tsetse flies, the endosymbiont *Wigglesworthia glossinidia* was shown to produce vitamin B-6 (pyridoxine) and elimination of the symbiont showed that *Wigglesworthia* influences immunity, reproduction and digestion^29^. A closer look at how tsetse flies utilized vitamin B-6 showed that it was critical for proline homeostasis, which tsetse flies rely on to produce ATP^30,31^. These metabolic downstream effects of vitamin provisioning could be present in bed bugs, requiring further investigation.

Furthermore, it remains to be determined whether dramatic effects on fecundity and hatch rate due to *Wb* elimination, are due exclusively to an effect on females, or whether reproduction is also affected in aposymbiotic males. It has been shown that the blood source fed to males can affect sperm competition and female fecundity^32^ but the effect of *Wb* removal on male reproductive fitness has not been investigated. Both sexes house *Wb* in gonad-associated bacteriomes^16^, so the elimination of *Wb* might uniquely affect reproductive traits in females and males. To date, all experimental designs in bed bugs have allowed males and females to mate within each treatment (e.g., either *Wb*^+^ or *Wb*^–^). A cross-treatment design, involving reciprocal crosses of *Wb*^+^ × *Wb*^–^, should reveal sex-specific effects of *Wb*.

Finally, *Wb* has been investigated extensively for its ability to interfere with host reproduction through cytoplasmic incompatibility (CI)^13^ which is typically detected as a reduction in egg hatch rate^33^. CI expression in *C. lectularius* thus would result in unfertilized eggs, which would be resorbed, resulting in lower oviposition with minimal effects on egg hatch. It would be fascinating to discover whether *Wb*-free bed bugs experience fitness-related deficiencies in part due to CI.

In summary, we found ddPCR much more reliable than qPCR for detecting low copy numbers and confirming the absence of *Wb* in aposymbiotic bed bugs. We therefore urge a careful re-examination of other nutritional symbioses with ddPCR to confirm that the symbiont was fully eliminated. The results presented in this study not only highlight the nutritional importance of *Wb* in bed bugs but also provide a useful experimental design to examine other endosymbiont-host relationships and methods for confirming the elimination of a symbiont. It is possible that *Wb* is responsible for providing additional nutritional benefits that contribute to adult size and fecundity in bed bugs. The mechanisms of transfer of B-vitamins from *Wb* to its host and how B-vitamins influence development, fecundity and survival are still unknown and would be a valuable area for future investigations. In addition, elucidating male vs. female effects on fitness traits would be useful to understand how *Wb* influences bed bug success.

## Materials and methods

### Insects

We used the Harold Harlan strain of *C. lectularius*. This strain was collected in 1973 in Ft. Dix, NJ, USA, and reared on a human host. Since 2008 bed bugs were fed defibrinated rabbit blood at North Carolina State University using an artificial feeding apparatus. The stock colony was used for the *Wb-*present (*Wb*^+^) treatments, and a second colony was maintained on antibiotic- and vitamin-supplemented blood to eliminate *Wb*, generating a *Wb*-absent (*Wb*^–^) lineage of *C. lectularius*. All colony bed bugs, experimental nymphs, adults and eggs were maintained in the same incubator at 27 °C, a 12:12 (L:D) photoperiod, and 45 ± 5% RH, and fed using the same methods. For all treatments, and colony maintenance, bed bugs were fed defibrinated rabbit blood (Hemostat Laboratories, Dixon, CA) through a custom-made glass feeder (Prism Research Glass, Raleigh, NC), water-jacketed and warmed to 35℃ using a thermal circulator water bath (B. Braun Biotech, Inc., Allentown, PA), as previously described^34^.

### Generating a *Wolbachia*-free (*Wb*^–^) colony

Two hundred adult virgin females and 100 virgin males 7–10 days after emergence were taken from the stock colony, and placed in a round plastic container (41 mm diameter × 67 mm) with folded filter paper and a 0.3 mm-mesh screened lid through which bed bugs could feed (oviposition jar). Adults were fed for 1 h. Adults remained in the container post-feeding to mate and lay eggs for five days and then all adults were removed and the container with eggs was placed in an incubator for 10 days. Cohorts of fifty 7–10 day-old first instars were transferred to three round plastic containers with filter paper and 0.3 mm-mesh screened lid for feeding (colony jar). Blood was fortified with 10 µl (= 10 µg) of a rifampicin-PBS solution (1 mg rifampicin/1 ml PBS) and 10 µl of an undiluted Kao and Michayluk vitamin solution (K3129, 100-fold concentration of vitamins, Millipore Sigma) per ml blood; generally, 2 ml of blood were provided to each treatment in each feeding session. The fortified blood was fed weekly to nymphs throughout development to rear the *Wb*^–^ colony. The colony was maintained for 15 months (May 2019 to Sept 2020) on weekly rifampicin- and vitamin-supplemented blood (#2 in Fig. 1). To mitigate any side effects of antibiotic treatment, a subset of adults was removed from the rifampicin treatment, placed in a new colony jar and reared for one generation before we initiated the experiments. These adults were fed vitamin-supplemented blood without rifampicin to produce a new “antibiotic-free *Wb*^–^ generation” (#3 in Fig. 1), and were the parental stock for the experimental bed bugs. Virgin females (100) and virgin males (50) 7–10 days after adult emergence, from the antibiotic-free *Wb*^–^ colony, were placed in a fresh oviposition jar and fed vitamin-supplemented blood for 1 h. Adults remained in the jar to mate and oviposit for 5 days and then removed. The first instars that hatched were used for the *Wb*^–^ treatments (#4 in Fig. 1). For *Wb*^+^ treatments, a second oviposition jar with the stock colony of the Harold Harlan strain was reared in parallel using the same methods, but the bed bugs were fed blood without vitamin-supplementation, to generate *Wb*^+^ first instars for the study (#5 in Fig. 1). The stock colony was never treated with antibiotics and never received vitamin supplementation.

### DNA extraction

To confirm the *Wb* status in bed bugs of the *Wb*-free colony, adult females (*n* = 13) from the rifampicin treatment (*Wb*^*–*^) and nymphs (*n* = 13) and adults (*n* = 20) from antibiotic-free *Wb*^–^ generation were assayed by ddPCR for the presence of *Wb*. We sampled adults from the *Wb*^–^ colony before the rifampicin treatment was discontinued (#2 in Fig. 1) and analyzed for the presence of *Wb*. We also sampled the nymphs and adults that emerged from the first rifampicin-free generation (*Wb*^–^Vit^+^; #3 in Fig. 1) and tested for *Wb* prior to experimentation (ddPCR in Fig. 1). Then, during the experimentation period, and throughout nymph development we sampled one third instar (*n* = 5), one fifth instar (*n* = 5), one adult male (*n* = 5) and one adult female (*n* = 5) from each replicate vial in all four treatment groups for *Wb* quantification to confirm that experimental bed bugs remained *Wb*-free.

Bed bug samples were individually surface sterilized with 10% bleach and 70% ethanol, followed by three washes with sterile water. The rationale was to eliminate potential DNA contamination from contact with feces, exuviae, and dead bugs. The samples were homogenized for 60 s using the FastPrep-24 tissue homogenizer (speed: 4 m/s; MP Biomedicals, Solon, OH) followed by DNA extraction using the DNeasy Blood and Tissue kit (Qiagen, Germantown, MD). Briefly, the homogenized samples were incubated at 56 °C in 180 µl of ATL buffer and 20 µl of proteinase K mixture for 4 h. Samples were briefly vortexed, 200 µl of AL buffer was added, and the samples were incubated for 10 min at 56 °C. The samples were then mixed with 200 µl of absolute ethanol and centrifuged through spin columns. The spin columns were washed with AW1 and AW2, following the manufacturer’s protocol, and DNA was eluted in sterile nuclease-free water. Following the elution, the samples were further purified, concentrated by ethanol precipitation, reconstituted in 100 µl sterile nuclease-free water, and stored at – 20 °C until further use. All the samples were amplified for *Wb* and total bacteria targeting *wsp* and 16S rRNA genes respectively, and a subset of samples were amplified for bed bug elongation factor ef1α gene using conventional PCR and agarose gel electrophoresis (Supplementary methods).

### Quantification of *Wolbachia*

Droplet digital PCR (ddPCR) was performed on individual bed bug genomic DNA samples for the absolute quantification of *Wb* in the four treatment groups. All adults and nymphs used for *Wb* quantification were newly molted and unfed. Each sample was quantified in duplicate on separate days. The ddPCR was conducted in a 20 µl reaction mixture comprising of 10 µl 2 × QX200 ddPCR EvaGreen master mix (Bio-Rad Laboratories, Hercules, CA), wsp-F (5′-CCGTATGTTGGCATTGGTGT-3′) and wsp-R (5′-AAGCTAGCGCCATAAGAGCC-3′) primers (final concentration: 250 nM), 4 µl of gDNA, and 0.25 µl (10 units) CviQI restriction enzyme. The primers designed for this study target a 182 bp region of the *Wb* surface protein gene *wsp* (NCBI repository, Accession AP013028.1, see Supplementary Information). The reaction mixture and 70 μl of droplet generation oil for EvaGreen (Bio-Rad) were loaded into the respective wells of a DG8 disposable cartridge (Bio-Rad) and droplets were generated with the QX200 droplet generator (Bio-Rad). The 40 µl emulsified samples were then transferred to a 96 well plate, sealed and underwent amplification in a T-100 Thermal Cycler (Bio-Rad). A positive control comprising gDNA from surface sterilized bed bug eggs, as well as a no-template control (NTC), were included in every PCR run. The thermocycling conditions were 1 cycle of 5 min enzyme activation at 95 °C, followed by 40 cycles of 30 s denaturation step at 95 °C, and 60 s at 58 °C for annealing and extension, followed by one cycle of 5 min at 4 °C and 10 min at 90 °C for droplet stabilization, and a final hold at 4 °C. The temperature ramp rate was set to 2 °C/s, with the lid heated to 105 °C, following Bio-Rad recommendations. Post thermocycling, the 96-well PCR plate was loaded into a QX200 droplet reader (Bio-Rad), which reads the droplets from each well of the plate. Analysis of the ddPCR data was performed with QuantaSoft software version 1.6 (Bio-Rad). Our lower limit of detection was 0.5 copies of the *wsp* gene of *Wb* per µl.

### Treatment groups: nymph development and adult emergence

Figure 1 shows the experimental design. Twenty 3–5 day-old first instars from the antibiotic-free *Wb*^–^ colony, were transferred to each of five 20 ml vials for the *Wb*^–^ without vitamin-supplementation treatment group (*Wb*^–^Vit^–^) and five additional vials for the *Wb*^–^ with vitamin-supplementation treatment group (*Wb*^–^Vit^+^) (#4a in Fig. 1). Twenty 3–5 day-old first instars from the untreated stock colony were transferred to each of five 20 ml vials for the *Wb*^+^Vit^–^ treatment group (*Wb*-present, no vitamins added) and five additional vials for the *Wb*^+^Vit^+^ treatment group (*Wb*-present, vitamins added) (#4b in Fig. 1). Thus, each of the four treatments started with 100 first instar bed bugs and all bed bugs were fed weekly. The *Wb*^+^Vit^+^ and *Wb*^–^Vit^+^ treatments were fed vitamin-supplemented blood with undiluted Kao and Michayluk vitamin solution (10 µl/ml blood), and the *Wb*^+^Vit^–^ and *Wb*^–^Vit^–^ treatments were fed blood without additional vitamins. Vials were placed individually on an artificial feeder for 20 min and the numbers of fed, unfed and dead nymphs were recorded after each feeding for 16 weeks. Newly emerged females and males were removed and paired in the adult fecundity assays, below. The percentage nymphs that fed, cumulative adults that emerged and cumulative dead nymphs were calculated for each vial (replicate) of 20 nymphs (*n* = 5) per treatment.

### Adult fecundity, female survivorship and body size

When adults began to emerge in the nymph development assays, they were removed and paired with an adult of the opposite sex from the same treatment group (#5 in Fig. 1). All the females that emerged from each treatment group were used, but the number of females in each treatment varied. Each pair was placed in a 7.5 ml vial with a strip of filter paper for egg laying and a screened cap for feeding. The filter paper contacted the screen to ensure access to the blood. Paired males and females were fed for 20 min the same blood treatment as their respective nymphal treatment group. If either the male or female was not fully engorged, the vial was placed back on the feeder until both adults were fully engorged. After feeding, vials were placed in the incubator and after 5 days, the male was removed and placed in a 20 ml vial with other males from the same treatment group, and fed weekly. The female was moved to a new 7.5 ml vial and fed individually every 5 days for 45 days to assess egg laying of each female. Females were remated individually on day 20. Vials containing eggs were stored in an incubator for 10 days, and then moved to a – 20 °C freezer to preserve eggs and hatched first instars for counting. Eggs and first instars were counted using a dissecting microscope (MZ6, Leica microscopes, Feasterville, PA). The number of eggs and egg hatch per female during 45 days was calculated for each treatment group (*Wb*^+^Vit^–^: *n* = 24, *Wb*^+^Vit^+^: *n* = 24, *Wb*^–^Vit^–^: *n* = 23, *Wb*^–^Vit^+^: *n* = 15). All adults were placed in a – 20 °C freezer after 45 days and the size of the thorax was measured using a digital microscope (Andonstar AD409, Shenzhen, China). If a female died before 45 days, the date was recorded and used to assess survivorship and the female was placed in a – 20 °C freezer for measurements.

### Comparison of vitamin mixes

Newly emerged adults from the *Wb*^+^ and *Wb*^–^ colonies were paired and fed blood supplemented with either the Lake and Friend (L&F) total vitamin mix^35^, which has been used in similar studies^16,18,31^ or the Kao and Michayluk (K&M) vitamin solution, which we have previously used^27,28^. The K&M and L&F vitamin concentrations used in 1 ml of blood can be found in Supplementary Information. Twenty *Wb*^+^ and 20 *Wb*^–^ pairs were placed pairwise in 7.5 ml vials with filter paper for egg laying and screened caps for feeding. The pairs were fed and used only if both sexes were fully engorged after the first feeding. The male was removed after 5 days, and the female was moved to a new vial and fed every 5 days for 15 days. The eggs from each vial were placed in an incubator for 10 days to hatch, then placed in a – 20 °C freezer. Egg number and percent hatch were recorded for each pair. We conducted this comparison for only 15 days to avoid the subsequent decline in oviposition in *Wb*^–^ females. The results from bed bugs fed the L&F vitamin mix were compared to similarly treated adult pairs that were fed the K&M vitamin solution during the adult fecundity assay.

### Data analysis

All analyses were conducted using JMP version 13 (SAS Institute, Cary, NC). ANOVA was used to compare vitamin mixes and male and female thorax width. If the ANOVA results were significant, Tukey’s HSD test was used to detect differences among treatments. Prior to using an ANOVA, the data were tested for normal distribution using the Shapiro–Wilk W test and for homogeneity using the O’Brien test, and percent data were arcsine square root prior to analysis.

A product-limit (Kaplan–Meier) survival analysis was conducted to examine the distribution of adult emergence and adult survival. The Wilcoxon Chi-square statistic test was used to determine differences among treatments. Repeated measures ANOVA was used to assess the percentage nymphs that fed, cumulative adult emergence, cumulative mortality, total eggs per female and percent egg hatch per female. The *F*-test was used to detect differences among treatments, and the Wilk’s Lambda multivariate test was used to assess differences over time when Sphericity Chi-squared was not significant. If the Sphericity test was significant, then the univariate test G–G Epsilon was used to determine whether there was a significant difference over time. We present summary data as box plots, showing all the replicates, as well as the mean value (white circle with a white horizontal line). The box represents the interquartile interval (50% of the replicates) and the black horizontal line within the box is the median. Whiskers indicate the 10th and 90th percentiles.

## Supplementary Information

Supplementary Figure S1.

## Data Availability

The data supporting the findings in this study and the *wsp* sequence corresponding to Accession AP013028.1 in the NCBI repository are in the Supplementary Information.
