# Supplementary material for: Effects of Wolbachia elimination and B-vitamin supplementation on bed bug development and reproduction
Source: Sci Rep. 2022 Jun 17;12:10270. doi: 10.1038/s41598-022-14505-2 (PMC9205976; doi:10.1038/s41598-022-14505-2)
Supplement: Supplementary file 1 — Supplementary Figure S1. [file 41598_2022_14505_MOESM1_ESM.pdf]

Supplementary Information for

Scientific Reports

Effects of *Wolbachia* elimination and B-vitamin supplementation on bed bug development and reproduction

Mauri L. Hickin[1*], Madhavi L. Kakumanu[1], Coby Schal[1*]

[1]Department of Entomology and Plant Pathology, North Carolina State University, Raleigh, North Carolina, USA

*Authors for correspondence:

Mauri L. Hickin – Email: mlhickin@ncsu.edu

Coby Schal – Email: coby@ncsu.edu

Supplementary Methods:

The genomic DNA of all the samples was amplified for the bacterial 16S rRNA gene using universal 27F (5′-AGAGTTTGATCMTGGCTCAG-3′) and 1492R (5′-GGTTACCTTGTTAC GACTT-3′) primers[1], and the *Wolbachia wsp* gene using wsp-F (5'-CGTATGTTG GCATTGGTGT-3') and wsp-R (5'-AAGCTAGCGCCATAAGAGCC-3') primers (this study). A subset of samples was amplified for the bed bug elongation factor 1α (ef1α) gene using CLefF (5′-GCAAATGCCTTATTGAAGCTCTC-3′) and CLefR (5′-GGAAGCCTAAGAGGCTTGT CAG-3′) primers[2]. The PCRs were conducted in a 12 µl reaction mix comprising 6 µl of AmpliTaq Gold 360 2X Master mix (Thermo Fisher), 0.6 µl of 10 µM of each primer, and 2 µl of DNA. A no-template control and positive control (DNA from bed bug eggs as a template for 16S rRNA and *wsp genes* and DNA from bed bug legs for ef1α gene) was included in every PCR run. The PCR protocol for amplification of the 3 gene targets was as follows:

16S rRNA gene: initial activation at 95°C for 10 min followed by 35 cycles of 94°C for 30 s, 50°C for 30 s, and 72°C for 1:30 min, and a final extension at 72°C for 10 min.

wsp gene: initial activation at 95°C for 10 min followed by 40 cycles of 95°C for 30 s, 58°C for 30 s, and 72°C for 1 min, and a final extension at 72°C for 5 min.

ef1α gene: initial activation at 95°C for 10 min followed by 35 cycles of 95°C for 15 s, 55°C for 15 s, and 72°C for 30 s and a final extension at 72°C for 5 min.

The amplified products were verified on 1.2% agarose gels.

Supplementary References:

1 Lane, D. 16S/23S rRNA sequencing, *In Nucleic acid techniques in bacterial systematics* (eds. Stackebrandt, E., Goodfellow, M). 115–175 (John Wiley and Sons, 1991).

2 Hosokawa, T., Koga, R., Kikuchi, Y., Meng, X. Y. & Fukatsu, T. *Wolbachia* as a bacteriocyte-associated nutritional mutualist. *Proc. Natl. Acad. Sci. U.S.A.* **107**, 769-774 (2010).

Supplementary Figure S1

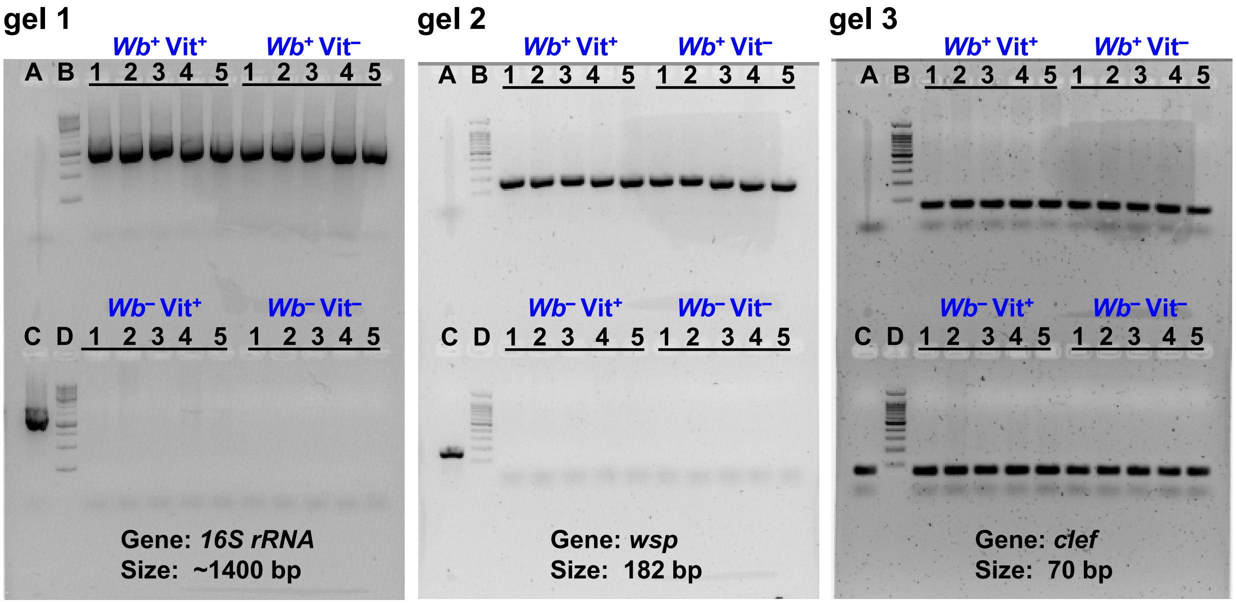

Figure S1. Agarose gel electrophoresis of the amplified PCR products of genomic DNA of 5th instars from 4 treatments targeting 3 genes. gel 1: 16S rRNA gene (total bacteria); gel 2: *wsp* gene (*Wolbachia*); gel 3: *clef* gene (bed bug DNA). In all gels, lane A: No template control; lanes B and D: molecular markers; lane C: Positive control; lanes 1–5: samples representing 5 replicates each of the 4 treatments indicated in blue. Wb^+ and Wb^- represent with-*Wolbachia* and without-*Wolbachia*, respectively. Vit^+ and Vit^- represent with-Vitamins added and without-Vitamins added, respectively.
